# Celecoxib accelerates functional recovery after sciatic nerve crush in the rat

**DOI:** 10.1186/1749-7221-3-25

**Published:** 2008-11-26

**Authors:** Carlos R Cámara-Lemarroy, Francisco J Guzmán-de la Garza, Ernesto A Barrera-Oranday, Andrés J Cabello-García, Armando García-Tamez, Nancy E Fernández-Garza

**Affiliations:** 1 Department of Physiology, Universidad Autonoma de Nuevo Leon, School of Medicine, Av. Francisco I. Madero y Dr. Eduardo Aguirre Pequeño S/N.o, Col. Mitras Centro, 64460, Monterrey, Nuevo Leon, Mexico

## Abstract

The inflammatory response appears to be essential in the modulation of the degeneration and regeneration process after peripheral nerve injury. In injured nerves, cyclooxygenase-2 (COX-2) is strongly upregulated around the injury site, possibly playing a role in the regulation of the inflammatory response. In this study we investigated the effect of celecoxib, a COX-2 inhibitor, on functional recovery after sciatic nerve crush in rats. Unilateral sciatic nerve crush injury was performed on 10 male Wistar rats. Animals on the experimental group (n = 5) received celecoxib (10 mg/kg ip) immediately before the crush injury and daily for 7 days after the injury. Control group (n = 5) received normal saline at equal regimen. A sham group (n = 5), where sciatic nerve was exposed but not crushed, was also evaluated. Functional recovery was then assessed by calculating the sciatic functional index (SFI) on days 0,1,7,14 and 21 in all groups, and registering the day of motor and walking onset. In comparison with control group, celecoxib treatment (experimental group) had significant beneficial effects on SFI, with a significantly better score on day 7. Anti-inflammatory drug celecoxib should be considered in the treatment of peripheral nerve injuries, but further studies are needed to explain the mechanism of its neuroprotective effects.

## Findings

After injury to peripheral nerves, a sequential pattern of axonal degeneration and myelin degradation, followed by rapid regeneration, is initiated [1]. The inflammatory process and its mediators have been implicated in the regulation of both the axonal degenerative and regenerative processes after injury [2,3]. One of the inflammatory mediators that might play an important role during these processes is the enzyme cyclooxygenase-2 (COX-2), responsible for metabolizing cell membrane arachidonic acid into prostaglandins, among other pro-inflammatory effects [4].

COX-2 is upregulated and prostaglandin production increased in macrophages and Schwann cells, after various types of peripheral nerve injury [5,6]. Studies on the upregulation of COX-2 during axonal regeneration have mainly concentrated on its participation in the induction of neuropathic pain, instead of on the regeneration process itself [7]. However, the fact that COX-2 is so strongly upregulated after nerve injury, and also able to modulate inflammatory mediators such as pro-inflammatory cytokines, suggest an important role for this enzyme in the evolution of nerve regeneration as well [8].

Celecoxib (CLX) is a selective COX-2 inhibitor with potent anti-inflammatory and analgesic properties [9]. CLX has shown neuroprotective properties in models of cerebral ischemia and experimental inflammatory neuritis [10,11]. The use of CLX has also been shown to be effective in reducing neuropathic pain following peripheral nerve injury in rats [7]. A recent study found that acetyl salicylic acid, a non-selective COX inhibitor, could accelerate functional recovery after a nerve crush lesion in the rat, although other mechanisms of action were believed to be involved [12]. However, the effects of CLX on functional recovery after peripheral nerve injury, to the best of our knowledge, have not been studied before. In this study we investigated the effects of CLX on functional recovery following peripheral nerve injury using a rat sciatic nerve crush model.

Animal procedures were performed in accordance with the proper use and care of laboratory animals. In this study15 male Wistar rats (250–300 g) were used. The animals were kept in a temperature controlled room, on a 12-hour light/dark cycle, with access to food and water *ad libitum*.

The animals were randomly assigned into 3 different groups: Experimental (n = 5), Control (n = 5) and Sham group (n = 5). In experimental and control groups, unilateral sciatic nerve crush was done as follows: After anesthesia with pentobarbital (Anestesal, Pfizer Inc, Mexico) (50 mg/kg ip), a small incision is made in the upper tight, and muscles are separated in order to expose the sciatic nerve. The nerves are then crushed with a 1 mm wide non-serrated hemostatic clamp at a standardized force at mid-tight level for 30 seconds. In sham group the sciatic nerve was exposed but not crushed. Afterwards muscle and skin are sutured separately, and the animal left to recuperate in individual cages. This crush injury can be used as a model of axonotmesis to study nerve function recovery [13].

Animals in the experimental group received CLX (Celebrex^®^, Pfizer Inc, Mexico) (10 mg/kg ip) immediately before and daily for 7 days after the injury. Control group received normal saline at the same time periods.

Evaluation of the sciatic functional index (SFI), a reliable assessment of nerve recovery [14], was carried out in all animals in days 0 (before surgery) and on days 1, 7, 14 and 21 after surgery. The hind feet of the rats was marked with water soluble black ink, and then the animal was allowed to walk freely across a 12 cm wide and 50 cm long confined walkway leaving its foot prints on a white piece of paper covering the walkway floor. The distance from the heel to tip of the third toe and both the toe-spread and intermediary toe-spread, defined as the distance between the first and fifth, and between the second and fourth toe, respectively, was measured to the nearest 0.5 mm. Both normal and injured limbs were evaluated. We measured 3 footprints per limb per animal were and then averaged the values in order to calculate the SFI for each animal, using a formula described elsewhere [15]. An SFI nearing 100 indicates severe impairment whereas an SFI nearing 0 is considered normal.

Functional recovery was also examined by registering the day of motor and walking onset in all animals as described by Gold et al [16]. Two blinded observers examine recovery daily, and register the number of days that each animal takes in order to be able to right the foot and move its toes (motor onset), and to walk using the foot and toes of the injured hindlimb (walking onset). The values are then averaged in order to calculate the mean for each group, and compared.

Changes in SFI over time were compared between groups using a 2-way repeated measures analysis of variance (ANOVA). When the ANOVA showed significant difference between groups, we applied a Tukey post hoc test to find the location of the difference. One-way ANOVA was used to compare motor and walking onset between control and experimental group. Data were analyzed with SPSS 11.0 (SPSS Inc. Software, Chicago, Illinois, USA) statistical software, and all values are expressed as mean +/- SD and *P *< 0.05 was considered statistically significant.

Before surgery, all animals had SFI values nearing 0 (normal), and immediately after nerve crush values above 90 (severely impaired), with no statistical difference between groups. Thereafter, starting from day 1 and until the last day of the study, rats in the experimental group showed significantly faster recovery compared with the control group (*P *= 0.02, between subjects repeated measures ANOVA), with post hoc tests showing a significant difference on day 7 (80.2 +/- 6.3 *vs. *66 +/- 12.1; *P *= 0.04) The motor and walking day onset was reached earlier in rats in the experimental group compared with control group, being statistically significant only for motor onset (11.4 +/- 1.1 *vs. *13.6 +/- 1.8). The Sham group had normal SFI values throughout the study (table 1).

**Table 1:** Motor functional recovery after sciatic nerve crush

Groups (n = 5 each)	SFI day 0	SFI day 1	SFI day 7	SFI day 14	SFI day 21	Motor Onset (days)	Walking Onset(days)
Control (saline)	-3.15 +/- 6.38	84.5 +/-11.8	80.2 +/- 6.3	59 +/- 10.2	27.5 +/-6.3	13.6 +/- 1.5	14.6 +/- 1.8

ExperimentalCelecoxib 10 mg/kg/day	0.83 +/- 6.64	77.9 +/- 3.3	* 66 +/- 12.1	52.4 +/- 8.3	16.1 +/-14.3	*11.4 +/- 1.1	12.8 +/- 1.3

Sham	0.14 +/- 5.88	3 +/- 3.7	-0.3 +/- 6.1	2.4 +/- 5.7	1 +/- 5.7	1	1

* indicates significant difference from control (*P *< 0.05)

All values are expressed as mean +/- SD.

Our results show that CLX can accelerate functional recovery following sciatic nerve crush in the rat. However, our small sample size and large SD are statistical weaknesses of our study, and the absence of histological or electrophysiological evidence prevents us of concluding that CLX has similar effects over axonal regeneration. COX-2 has been shown to modulate neuroinflammation in various neurological disorders [17] and pro-inflammatory cytokines have been implicated in the orchestration of the inflammatory process that lead to degeneration and regeneration after nerve injury [18]. One of these cytokines, Tumor necrosis factor alpha (TNF-alpha), is known to induce nuclear factor kappab (NF-kappab) activation, a transcription factor that promotes further pro-inflammatory cytokine production in inflammatory cells [19]. Recent evidence has shown that CLX can inhibit TNF-alpha dependent activation of NF-kappaB, resulting in strong anti-inflammatory activity [20]. Macrophages and Schwann cells also play essential roles in axonal degeneration and regeneration in injured peripheral nerves [21] and COX-2 is upregulated in both of these cells during peripheral nerve inflammation [22]. Additionally, COX-2 inhibition has been shown to inhibit macrophage and glial cell neurotoxic activity in neurons *in vitro *[23]. Whether one of these effects is the mechanism responsible for the results of our study requires further investigations. Nevertheless, our results suggest that CLX can beneficially alter the course of functional recovery after peripheral nerve injury.

## Abbreviations

COX-2: cyclooxygenase-2; SFI: sciatic functional index; CLX: celecoxib; TNF-alpha: tumour necrosis factor alpha; NF-kappaB: nuclear factor kappa b; SD: standard deviation.

## Competing interests

The authors declare that they have no competing interests.

## Authors' contributions

CRCL designed the study, performed research, and wrote the manuscript. FJGG helped design research and analyzed the data. EABO helped design the study and perform research. AJCG carried out the behavioral studies on the animals. AGT carried out the behavioral studies on the animals. NEFG analyzed data and revised the manuscript. All authors read and approved the manuscript.
